# Experiences of and attitudes towards HIV testing for Asian, Black and Latin American men who have sex with men (MSM) in the SELPHI (HIV Self‐Testing Public Health Intervention) randomized controlled trial in England and Wales: implications for HIV self-testing

**DOI:** 10.1186/s12889-022-13189-7

**Published:** 2022-04-22

**Authors:** Emily Jay Nicholls, Phil Samba, Leanne McCabe, Mitzy Gafos, Andrew N. Philips, Roy Trevelion, Alison J. Rodger, Fiona M. Burns, Peter Weatherburn, T. Charles Witzel

**Affiliations:** 1 Department of Public Health, Environment and Society, London School of Hygiene and Tropical Medicine, 5-17 Tavistock Place, London, WC1H 9SH UK; 2 Institute for Global Health, University College London, Royal Free Hospital, Rowland Hill Street, London, NW3 2PF UK; 3 Medical Research Council Clinical Trials Unit at University College London, 90 High Holborn, London, WC1V 6LJ UK; 4 Department of Global Health and Development, London School of Hygiene and Tropical Medicine, 5-17 Tavistock Place, London, WC1H 9SH UK; 5 HIV I-Base, 107 The Maltings, 169 Tower Bridge Road, London, SE1 3LJ UK

**Keywords:** HIV, HIV self-testing, Ethnicity, Men who have sex with men, Randomised controlled trial, New prevention technologies

## Abstract

**Background:**

HIV self-testing (HIVST) could play an important role in improving access to testing and therefore reducing inequalities related to late diagnosis of HIV, while also improving access to HIV prevention interventions such as HIV pre-exposure prophylaxis. This study sought to understand the potential role of HIVST by exploring the experiences of Asian, Black and Latin American men who have sex with men (MSM) accessing the gay scene and the circulation of HIV testing norms; experiences of accessing HIV testing services; HIVST acceptability and preferences for intervention adaptations.

**Methods:**

Twenty-nine qualitative interviews were conducted with Asian, Black and Latin American MSM who had participated in SELPHI, an HIVST randomised controlled trial. Topics included HIV testing history, HIV testing patterns, experiences of accessing sexual health services, mental health, engagement with HIVST and SELPHI, and experiences of the gay scene. Interviews were audio recorded, transcribed and then analysed using a thematic framework.

**Results:**

The gay scene was identified as an important site for learning about HIV and being exposed to norms reinforcing the importance of protective behaviours. However, experiences of discomfort due to perceptions of ‘whiteness’ on the scene or experiences of racism may hinder the protective function the scene could play in developing norms influencing HIV testing behaviour. Discomfort in clinic waiting rooms was identified as a substantial barrier to accessing clinical services and many interviewees expressed preferences regarding the personal characteristics of healthcare providers. HIVST was found to be acceptable and some interviewees suggested potential adaptations of the HIVST offer, such as packaging HIVST with at home sexually transmitted infections testing options.

**Conclusions:**

HIVST responds to some service access barriers experienced by Asian, Black and Latin American MSM. The decoupling of HIV testing and clinic attendance may be particularly valuable for MSM of minority ethnic backgrounds who are likely to experience anxiety and discomfort in clinic waiting rooms more acutely than White MSM due to concerns around implied disclosure. This suggests that HIVST may have the potential to increase testing uptake and frequency, particularly for those with complex relationships with clinical services.

**Trial Registration:**

SELPHI was prospectively registered with the ISRCTN (ref: ISRCTN 20312003).

## Introduction

HIV remains a major public health issue in the UK, one which is increasingly a reflection of entrenched health inequalities among men who have sex with men (MSM). Although recent years have seen a significant reduction in HIV incidence in MSM as a whole, this decline has not been spread evenly across groups [1, 2].

MSM of White ethnicity born in the UK have seen the steepest decline in HIV incidence [2, 3]. MSM of Latin American ethnicities have seen less rapid falls compared with their White counterparts and Asian MSM continue to have higher rates of late diagnosis [1, 2]. The proportion of annual new diagnoses among those who had emigrated from Latin American countries rose from 2.8% in 2008, to 5.5% in 2017 [4]. A large proportion of Latin Americans diagnosed with HIV in the UK between these years were living in London and most were MSM [4]. Black MSM face additional barriers in accessing HIV testing and prevention opportunities including those related to staff perceived as being from similar cultural backgrounds and also racist conceptualisations of hypersexuality [5–7]. In addition, despite having comparable levels of sexual risk compared to other groups, Black MSM are more likely to test positive for HIV [1, 8–10].

Improving access to HIV testing may help reduce inequalities related to late diagnosis of HIV, while also improving access to HIV prevention interventions such as HIV Pre-Exposure Prophylaxis (PrEP). There is a clear need to address the specific barriers that MSM of minority ethnic backgrounds face in accessing HIV testing services. It is also important to determine whether HIV self-testing (HIVST) might have a role in improving access to testing for these groups, thereby reducing health inequalities.

There is a lack of literature focusing on the experiences of these groups in relation to HIV testing and other HIV prevention technologies within the UK and the rest of Europe, although there are some exceptions (for example on PrEP [5, 6]). For the most part, studies across Europe on HIV prevention technologies have tended to focus either on the experiences of gay, bisexual and other MSM, of people of minority ethnic backgrounds [11, 12] and/or of specific migrant groups [13] rather than the intersections between sexuality, ethnicity and migration. This elision is played out in the reporting of HIV testing rates, which also tend to focus on either MSM or disaggregate testing rates for heterosexuals by ethnicity (e.g. [14]).

Research on attitudes towards and experiences of HIV testing has highlighted significant concerns around privacy and confidentiality for those of minority ethnic backgrounds [11] and/or experiences of migration [12]. There is also some evidence to suggest that MSM of minority ethnic backgrounds face specific barriers to accessing HIV testing services [7, 15]. Research by McKeown et al. [16], for example, found that MSM of Indian, Pakistani and Bangladeshi ethnicity were more likely to be ‘very anxious’ about visiting a clinic than the other minority ethnic or White MSM surveyed. MSM from these South Asian backgrounds were also more likely to find the waiting area uncomfortable; and were more likely to worry about being overheard when speaking with the receptionist [16]. Research by Datta et al. [17] found that although MSM from all ethnic backgrounds (particularly younger men) might experience anxiety around attending a sexual health clinic, Black and Asian participants were particularly concerned about being identified by others in their communities.

HIVST is a relatively new intervention whereby the user collects their own sample, processes the test and interprets the result themselves [18]. This is different from HIV self-sampling (HIVSS), where a user collects a sample and then sends this to be processed in a laboratory [19]. Initially banned in the UK because of concerns over the potential psychological impact of self-diagnosis, HIVST was legalised in 2014 and became commercially available in 2015 [20]. HIVST has benefits for marginalised groups by providing an additional convenient, private testing modality which can be delivered through a variety of mechanisms depending on the preferences of the intended beneficiaries [21]. HIVST has been shown to increase HIV testing frequency, and overall yield of positive results in MSM and may play a role in reducing health inequalities by overcoming some barriers to testing that are especially common among certain minority groups [22–24].

SELPHI (An HIV Self-Testing Public Health Intervention) was an online randomised controlled trial (RCT) of HIVST which recruited 10,111 MSM (cis and trans) who reported lifetime anal sex with men [25, 26]. It had two randomisations and two HIVST interventions. First the initial sample was randomised 60/40 to baseline HIVST (BT) vs standard of care (no baseline testing (nBT)); second at 3-months eligible men (remained HIV negative, reported condomless anal intercourse (CAI) and interest in further HIVST) who had received a baseline test were randomised 50/50 to 3-monthly offer of repeat HIVST (RT) vs standard of care (no repeat testing (nRT)). SELPHI also explored critical secondary outcomes including HIV testing uptake and frequency, sexually transmitted infections (STI) testing uptake and frequency, changes in volume of condomless anal intercourse (CAI) partners and experiences of harm [27].

This article aims to describe the experiences of and attitudes towards HIV testing for Asian, Black and Latin American MSM who took part in the SELPHI RCT in England and Wales, and the implications for HIV self-testing in these groups. We do this by exploring the relationship between participation in the gay scene, feelings of marginalisation and HIV testing norms; the experiences of Asian, Black and Latin American MSM accessing HIV testing services; and HIVST acceptability and preferences for intervention adaptations for these key groups.

## Methods

The SELPHI protocol is published elsewhere [25], as are comprehensive descriptions of the trial pathways and interventions [28–30].

For this qualitative sub-study, 29 interviews were conducted between April and July 2020 by EJN, PS and TCW; PS is a Black cis gay man, peer researcher and health promoter with an expertise in working with queer men of colour. EJN and TCW are White and they are a cis heterosexual woman and a cis gay man, respectively. All interviews were conducted remotely; 28 were conducted via video conferencing software, and one was conducted using instant messaging due to domestic privacy concerns.

Interviews were semi-structured and followed a topic guide which had been adapted from previous sub-studies to include additional relevant questions. The topic guide covered: HIV testing history, HIV testing patterns, experiences of sexual health services, mental health, engagement with HIVST and SELPHI, and experiences of the gay scene. This analysis focuses on all of these except mental health which will be reported separately.

Interviews were transcribed verbatim and the Framework Method [31, 32] was employed to analyse interview transcripts. EJN, PS and TCW familiarised themselves with the transcripts and developed an analytical framework. This was done by comparing emergent themes with those from previous qualitative work on the SELPHI study [28, 29] and adding and amending theme codes as necessary. EJN, PS, TCW, PW, AJR & FMB then met to discuss this framework and refined it. The framework was piloted on two transcripts by EJN and TCW and then further refined. The transcripts were the coded in tandem by EJN and PS and discussed throughout, along with TCW.

## Results

We interviewed 29 cis MSM from Asian, Black and Latin American backgrounds. The majority (*n* = 19) were under 35 years of age (Table 1). Thirteen described their ethnicity as Black (Black African, Black Caribbean or mixed), with fewer numbers of Asian (*n* = 11) and Latin American (*n* = 5) MSM. Most were gay and highly educated, although 12 had a medium or low level of education.

**Table 1:** Participant demographics

Participant demographics		
**Attribute**	Options	Count
**Age**	18–25	9
	26–35	10
	36–45	3
	46 +	6
**Ethnicity**	Black / Black Caribbean inc. mixed	8
	Black / Black African inc. mixed	5
	Asian inc. mixed	11
	Latin American	5
**Sexual Orientation**	Gay	22
	Bisexual	2
	Other / undisclosed	4
**Education**	Low^a^	2
	Medium^b^	10
	High^c^	16
**Last HIV test**	< 12 months	19
	> 12 months	7
	Never	2

^a^GCSEs and below

^b^A-levels or equivalent, higher education below degree level

^c^Degree or higher

The first section describes the relationship between the gay scene (online and offline) and the development of norms around HIV testing and sexual health, as well as experiences of racism on the scene. It forms the background and context to experiences of accessing HIV and sexual health services more broadly. The second section describes experiences of and barriers to HIV and sexual health services. The final section focusses on interviewees’ perceptions of the relevance and utility of HIVST for MSM of minority ethnic backgrounds as well as their suggested adaptations of the kit.

### Testing norms and relationships with the gay scene

This section describes experiences of marginalisation and exclusion that MSM of minority ethnic backgrounds face when engaging with the commercial gay scene in both offline and online spaces and the links they made between the gay scene, HIV testing and sexual health through peer interactions and exposure to sexual cultures. Although there are a wide variety of venues which could be said to encompass the gay scene, we focus in the analysis below primarily on experiences of accessing bars, clubs and online dating and hook-up apps.

Most interviewees had previously engaged with the scene by attending gay bars and clubs but had withdrawn as they had got older and ‘life’ had ‘got in the way’. A small proportion of interviewees described discomfort with the scene, resulting from a perception of it as privileging youth and as being overly focused on physical attractiveness. For some, this also included a perception of the gay scene as predominantly White and of the often-unacknowledged racism of White men on the scene:

‘When I’ve gone to certain places, it’s very... I think it’s very, very white and I don’t think gay men, some gay men, realise how racist they can be. Now, I’ve got a lot of White friends and gay friends. But I’ve sometimes not been very happy, I’ve been very sensitive when I’ve gone to certain places. It’s very hard to explain.’ PN 19, 46+ years old, Black Caribbean

When experiences of racism were identified and described, they tended to be in the form of sexual racism. This was most often described by Black men who were often viewed as hypersexual and especially masculine by White MSM:

‘So when you’re on the scene, it’s either that people see you [in terms of your ethnicity] or they don’t see you as that and then you become invisible. And it’s very hard to gauge if people are genuinely seeing you for who you are. And then there can be that side of it that makes you feel like you’re a kid in a candy shop, if you really take advantage of it. But I feel like that’s a bit soul-destroying as well because then you never really feel like you get anyone that actually sees you. Because everyone just wants... or they think they’re after one aspect of you. Or because they only date Black men. So you just become the next Black man on their arm, sort of thing.’ PN02, 46+ years old, Black Caribbean

Racism was also experienced in online gay spaces such as hook-up apps, generally taking the form of dismissive comments from other users or blanket statements on profiles indicating a lack of interest in entire ethnic groups. This was usually brushed off as being of little importance or understood as simply a matter of preference rather than a form of racism. However, a small number of interviewees also described abusive language or derogatory comments, also often underplayed by interviewees as being of little importance:

‘Dating apps you just have people that just go out of their way to send you a message just to be derogatory for no reason. So that’s the main ones […] And they, yes, are just quite dismissive and it could be the fact I’m Black is the thing they’ll use as a driving point or using derogatory language just because they want to spark, I don’t know, some anger or something, which I just block them ‘cause it’s online and they’re a stranger who means nothing to me.’ PN21, 18-25 years old, Black Caribbean

These perceptions and experiences are of particular importance as the gay scene was also described as a space to learn about HIV prevention and risk.

Participation in the scene underscored the importance of HIV testing norms as, for some, engagement with other MSM via the gay scene had been an important site for learning about HIV prevention and sexual health:

‘Before I started university, I guess I didn’t really know too much about it. I guess, I know why you would do testing but I didn’t really know. So why would you test every six months all different kind of things and obviously as I spent time in the gay scene it’s like, okay. So different people are telling me different things and I discovered about PEP and PrEP and stuff like that.’ PN18, 18-25 years old, Asian

For many MSM, observing or participating in the sexual cultures of the scene brought to the fore the importance of testing. However, norms were sometimes reinforced by identifying behaviours individuals found troubling, for example, a perception of people on the scene as being ‘promiscuous,’ highlighted the need to test regularly. For instance, when asked about whether his engagements with the gay scene had any influence on his perceptions of or attitudes towards HIV testing, PN03 below immediately recalled his shock when finding himself in a ‘dark room’ (a darkened room, sometimes located in a sex club or a night club where sex can take place):

‘[I was] completely shocked because you see people doing bareback and, like, sorry but people, like, bending over and, like, they don’t even look behind their backs. That makes me think, oh my god, does this person know about HIV, gonorrhoea, chlamydia and all the other things. So, it is these sort of things, certain situations that I know I have encountered. They make me think about HIV a lot more.’ PN03, 36-45 years old, Latin American

### Clinic experiences

This section explores the experiences of MSM of minority ethnic backgrounds when accessing sexual health clinics as well as their preferences for future service access. In particular we focus on the role of clinic waiting rooms and healthcare provider characteristics in shaping accessibility for HIV testing.

A large proportion of interviewees reported experiences of discomfort in the waiting rooms of sexual health clinics. There were no obvious differences between different ethnic backgrounds included in the study, although Asian men seemed the most likely to express discomfort, and Latin American men the least. This discomfort was due to a perception that other people in the waiting rooms were looking at them and judging them for being there:

‘I think also, I feel like the people at the GUM clinic are all… Not all of them but the ones that I’ve certainly, come across, are quite judgey and you feel like it’s a bad thing to be there. Even though I know that’s not the case, but you feel like when you’re in the GUM clinic, it’s because you’ve got a problem, rather than a preventative thing.’ PN13, 18-25 years old, Asian

Interviewees did not often relate these experiences to their ethnicity, however, a small number did. This was also identified by some interviewees as being related to their cultural background, and this was especially so for those interviewees who had migrated here from countries that they described as being conservative. This was not only discussed in relation to interviewees’ own experience, but also their perceptions of other minority ethnic MSM:

‘For a lot of people from Black and ethnic minorities, they often go outside of their area for testing. Because a lot of friends of mine, they wouldn’t get tested in their own area, for instance, for fear of seeing somebody because they’re not fully out.’ PN19 46+ years old, Black Caribbean

None of the Latin American interviewees described having any preference regarding the sexuality or ethnic background of their healthcare provider in general, with only one interviewee commenting that he would prefer a male healthcare worker if there was a need for swabs to be taken that were not self-administered. Despite the Latin American interviewees tending not to have a preference on the ethnic background of their healthcare providers, around a third of all participants did express a preference. Importantly, where comment was made on preference, this could also include an expression of ambivalence:

‘It can have double-edged swords. Sometimes you think, oh, they’re in your community. Will they judge you? But in general, it can be helpful […] I guess there’s that sense of familiarity, being able to feel a bit more secure. The person, if they’re a similar background, would maybe understand your cultural context a lot more and the challenges that you might face’ PN06, 46+ years old, Black Caribbean

Among Asian interviewees, there was a clear favouring of healthcare providers of a different ethnicity to themselves. Preferences were mixed for Black African and Black Caribbean men with regards to both the ethnicity and sexuality of their healthcare provider. For a small number of interviewees, age and perception of religious belief were also important factors, especially when coupled with ethnicity:

‘The older providers, who are of a similar ethnic background, maybe having a view of how I should be and then, it’s hard to pin it down as necessarily saying like it’s outright homophobia because it’s not like anything about, you know, it’s not like they’ve been, ‘You shouldn’t be gay’, or, ‘You shouldn’t engage in that’. It’s the kind of implicit, it’s not exactly this but like ‘go to church,’ and, you know? I guess some level of homophobia but very implicit.’ PN28, 18-25 years old, Black African

Overall, more interviewees expressed a preference for healthcare providers with a different ethnic background to themselves than those who expressed a preference for a provider with the same ethnic background. One Black African interviewee expressed a particular wariness of healthcare providers who were White gay men:

‘I feel like a straight White medical person will make more of an effort to understand or do research because they are not a minority. And I think a lot of gay White men think that they are a minority, so they don’t have to do the research, if that makes sense [...] So, I would actually trust a... Yes, I would trust anyone more than a gay White man, actually, I think’ PN26, 26-35 years old, Black African

### Experiences of using HIVST

In this section we explore themes regarding real and potential use of HIVST. Specifically, we begin by examining participants’ accounts of the role HIVST has in facilitating testing. We then discuss accounts of HIVST outcomes. Following this, we describe the perceptions of groups most likely to benefit from HIVST and potential intervention adaptations to increase accessibility and uptake among Asian, Black and Latin American MSM.

Interviewees reported positive experiences of participating in SELPHI and found the HIVST test kit to be well packaged and easy to use. Interviewees described a sense of autonomy over their own health and the ability to make decisions without healthcare worker oversight or having to justify their actions to a healthcare practitioner. This was a primary issue for those with concerns about healthcare providers and judgemental attitudes:

‘If I put myself at risk, fine, I can go and take my life into my hands and say, right, I want to get myself tested so I know where I sit. And what I don’t have to do is justify or explain my physical and emotional decisions to somebody else just so that I know whether I’m positive or not. And that, to me, is a hugely, hugely empowering action that we can take […] So that’s what I really love about the SELPHI testing kit. Is that I can do it without being judged.’ PN07, 46+ years old, Asian

When asked whether they would use HIVST alongside other services, such as continuing to access STI testing through sexual health clinics, responses were somewhat mixed. Some interviewees talked about the importance of ensuring that they were also testing for other STIs and said that they would continue to test for STIs at a clinic, with some also suggesting that they would use HIVST alongside clinic visits. Other interviewees suggested that HIVST would be likely to replace some clinic visits, and they would not be likely to attend for STI testing in the absence of symptoms or a sense that something was ‘not right,’ suggesting that HIVST may displace some routine STI testing.

The participant who received a positive result from his self-test did not initially believe the test outcome. He sought confirmatory testing within 4 to 5 days as advised, but expected this test to confer a negative result. In retrospect he recognised that subconsciously his primary motivation for seeking confirmatory testing may have been the understanding that he needed assistance in coming to terms with the result:

‘The clinic piece was very much a, “yes, I don’t believe in self-tests. I’m going get it all checked out while I’m here and just double check.” […] “And I’m not confident in my margin of error. I’ve given too much blood or whatever.” […] Maybe I actually went to the clinic to get me the support to help me understand or just hear it for myself. I think in that period, which I think was about four days, was just like as normal until they [sexual health clinic] told me otherwise.’ PN24, 26-35 years old, Asian

This individual described feeling isolated during the period following confirmatory testing as he was not open about his sexual orientation with his family and friends who were culturally conservative. He sought emotional support primarily through clinical services and from a private counsellor, only later discussing his sexual orientation and HIV status with a close friend. Although this was the only participant who tested positive in the sub-study, this is reassuring with regards to concerns about poor linkage to care, especially given that the participant also accessed a counsellor to emotionally process his diagnosis.

Overall, HIVST was felt to be an empowering intervention which led to increases in self-efficacy and provided the opportunity to test without accessing health services, more so for those who had complex relationships with sexual health clinics. There was indication that some individuals reduced their STI testing when they had access to repeat HIVST, but this was not universal. All felt they would seek confirmatory testing as recommended following a positive HIVST.

Interviewees offered a range of potential adaptations of the HIVST intervention. The most common suggestion was to package HIVST with other home STI testing options:

‘I would probably be happy to do it with everything else as well if there was a test for everything at home, that would be brilliant. Because going into a clinic and being in a room with other people in that situation is somewhat uncomfortable. And so the more of this kind of testing you can do for these kind of diseases at home, without societal and other impressions, the better, I think’ PN11, 26-35 years old, Black Caribbean

This would also ensure that those who did experience discomfort in accessing sexual health clinics were able to conveniently test for HIV and other STIs at the same time, an approach currently available with some HIVSS interventions.

When asked if there were any groups of people who they thought the test kit would be particularly useful for, several interviewees responded by saying that HIVST would be particularly useful for people of minority ethnic backgrounds. This was often related to a broader observation, identified by a number of interviewees, that people who valued discretion or found visiting sexual health clinics uncomfortable would find HIVST particularly useful:

‘Definitely people that are from backgrounds or communities that aren’t quite overt with such things or aren’t so able to head out and go to a clinic and be so open about it. I think it’s definitely beneficial for people that are a bit more discreet in what they’re doing or how they have to go about things, because of culture or because of anything like that. It’s very helpful as the way it pops through the post.’ PN21 18-25 years old, Black Caribbean

However, one interviewee was concerned that this may limit the potential for support from sexual health practitioners, as it would remove the need for contact with services which served a function beyond the test itself, especially around learning about sexual health. Although this view was only voiced by one interviewee, this may be an important issue for those who are not accessing information about HIV and sexual health elsewhere. For example, this may be particularly relevant for those who are also not engaged with the gay scene and as such are not benefitting from peer and community norms circulating within these spaces.

While HIVST was felt by several interviewees to be very useful for those who did not feel comfortable visiting sexual health clinics, two interviewees also highlighted that there may be barriers related to domestic privacy:

‘I stayed with my parents over the summer a few years ago. And I couldn’t do it then ‘cause I couldn’t let it go to my parents’ house. And also, I was moving house [last year] and I couldn’t do it then because I wasn’t sure when the test was going to arrive. I wasn’t absolutely sure when it was going to arrive within a week or whatever. And I didn’t want it to go to my old address.’ PN26 26-35 years old, Black African

Another suggested adaptation included an option where a user could order an HIVST online and then collect it. This was felt to be useful by several interviewees. That lack of access to privacy could be a potential barrier to accessing HIVST was also underscored during data collection, where one interview had to be conducted via instant messaging software as the interviewee had domestic privacy concerns and did not want to risk being overheard.

## Discussion

This qualitative study of 29 Asian, Black and Latin American MSM from across England and Wales examines key areas related to HIV testing for these priority groups. First, we described the ways men from minority ethnic backgrounds can feel excluded from the gay scene and explored the scene as a site where HIV testing norms and sexual health knowledge circulates. We also examined the experiences and preferences these groups have when accessing sexual health services, paying close attention to the role of waiting rooms and staff mix. Finally, we explored the impact of HIVST on changing HIV testing behaviour for these men, before examining the potential intervention adaptations which might facilitate increased uptake of this novel testing modality.

The analysis presented in this article delineates how the gay scene functions as a key site for health promotion and for the development of norms around HIV testing and sexual health [33, 34]. In accessing the scene MSM gain knowledge and are exposed to norms reinforcing the importance of protective behaviours, these norms are drawn both from peer interactions on the scene and also by identifying behaviours or dynamics within sexual cultures which prompt testing for MSM. Engagements with the gay scene are not only opportunities to gain knowledge about sexual health, they also form part of the broader backdrop and context to engagements with sexual health services. Therefore, the online and offline spaces that make up the gay scene may offer potential for the promotion and/or distribution of HIVST as an alternative testing modality, especially for those who face significant barriers to clinical services.

Our research, however, highlights how feeling unwelcome due to the ‘whiteness’ of the scene, or experiencing implicit or explicit racism or discrimination, may hinder the positive function the scene could play in developing norms influencing HIV testing behaviour. In line with previous studies, we found cause to suggest that marginalisation within the gay scene could lead to a decrease in contact with community norms highlighting testing, and potentially with health promotion initiatives [6]. As such, campaigns to promote HIV testing generally, and HIVST specifically, amongst MSM of minority ethnic backgrounds must account for the effects these dynamics on the scene might have for engagement. This is especially true when harnessing the scene for service delivery or provision.

A significant proportion of MSM interviewed in this sub-study experienced discomfort in accessing sexual health services, particularly in waiting rooms. This analysis suggests this discomfort may be particularly acute for those with cultural and religious backgrounds where sexuality and sexual health are disproportionately stigmatised. This is likely to be related to the possibility of being seen, or of friends or family finding out that they have been accessing these services and what this might mean. Previous work has also suggested that feelings of discomfort and a need for privacy and confidentiality may be particularly acute for MSM of minority ethnic backgrounds and for people of minority ethnic backgrounds of all sexualities [11, 12]. It should be noted that feelings of discomfort are experienced across ethnic backgrounds including White MSM [17] (not included in this sub-study), however this is likely to be exacerbated for some MSM of minority ethnic backgrounds. These findings echo work on PrEP acceptability for Black MSM, where interviewees suggested that a PrEP service may be more acceptable if situated outside of ‘traditionally ‘Black’ areas’ to ensure confidentiality from other community members, as well as work that has highlighted discomfort in waiting rooms for MSM from different ethnic backgrounds [5, 16].

Confidentiality and a non-judgemental approach are centrally important characteristics for clinic staff regardless of their personal characteristics. However, in line with previous research, we include discussion of preferences regarding healthcare providers’ ethnicity and sexuality in our analysis in order to describe our interviewees’ perceptions of how these characteristics shape providers’ attitudes and how this impacts and shapes accessibility [5]. This analysis paints a mixed picture with regards to the preferences of healthcare providers’ personal attributes, however, it does suggest that there are complex and multifaceted reasons why MSM across different ethnic backgrounds may have particular preferences regarding the personal attributes of healthcare providers. These include a desire for understanding (of cultural background, religion, or of sexuality) and concerns for either overt or implicit homophobia or judgement related to sexual orientation. This suggests significant complexity and a need for nuance in understanding the specific needs of minority ethnic MSM in clinical services, as well as the significant heterogeneity of experience and preference. Experiences of discrimination in sexual health settings were very rare, rather participants perceived the *potential* for discrimination from health care providers. Using markers of inclusivity (e.g. rainbow lanyards, posters highlighting diversity) in clinical services will provide signals to MSM from a range of backgrounds that services are inclusive and accepting.

Many elements of intervention acceptability for MSM from Asian, Black and Latin American backgrounds were similar to those found in other groups [28, 35]. Much of the high acceptability observed within this study came from removing HIV testing from clinical services and uncoupling it from staff. This increased accessibility for some MSM of minority ethnic backgrounds who had concerns about clinical services and was described as empowering. This echoes findings on the value of HIVST for other marginalised groups [35].

While some were concerned that HIVST may decrease engagement with STI testing, this was not universal. That such a significant proportion of interviewees experienced discomfort in waiting rooms also gives some indication of the potential HIVST might have for these groups, especially considering that one of the main adaptations suggested was to include tests for other STIs rather than making any significant changes to the offering itself.

### Strengths and limitations

This article presents a significant contribution to the knowledge base of experiences of minority ethnic MSM and issues relevant to HIV testing and sexual health in general, as well as HIVST in particular. However, there are some limitations to this study. Firstly, a significant proportion of interviewees were aged under 35. As the gay scene was described by some interviewees as privileging youth, it is likely that having a higher proportion of men over this age would have added depth to these accounts. Over half of the interviewees were also educated to degree level or above, which reflects broader trends of those with higher education levels being over-represented in research of this kind.

Although focusing on the experiences of Asian, Black and Latin American MSM has allowed for some comparison of trends between these groups, this has also meant that there have been relatively small numbers of interviewees representing each of the ethnicities included in this study. There are likely to be vast differences of experience and circumstance both within and between groups, which has implications for the generalisability of this work. Moreover, there are likely to be differences in experience according to whether interviewees were born in the UK or had migrated, with those in the latter group more likely to face significant and multiple barriers to accessing services. This did seem to be apparent in this study, but the small sample size made it difficult to make any strong claims based on migration status. As such, future work is needed to further develop the findings of this research.

Finally, when asked about their past experiences of HIV testing, participants described accessing different clinic types (including LGBT focussed clinics and those for the general public). Due to the small sample size and wide ranges of experience, we have not distinguished between different clinic types in this analysis.

## Conclusion

The findings of this sub-study suggest that HIVST can respond to some of the service access barriers experienced by Asian, Black and Latin American MSM. The decoupling of HIV testing and clinic attendance may be particularly valuable for MSM of minority ethnic backgrounds, who are likely to experience anxiety and discomfort in clinic waiting rooms more acutely than White MSM. HIVST was highly acceptable and no evidence was found that this intervention would decrease linkage to care. Future studies should focus on the effects of a history of migration on experiences of HIVST use and HIV testing more broadly.

## Data Availability

The dataset generated and analysed during the current study is not publicly available due the sensitive and personally identifiable nature of data concerning the experiences of a marginalised population, but is available from the corresponding author on reasonable request.

## Abbreviations

CAI: Condomless anal intercourse
HIVST: HIV self-testing
HIVSS: HIV self-sampling
MSM: Men who have sex with men
